# Ethics of stem cell‐derived gametes made in a dish: fertility for everyone?

**DOI:** 10.15252/emmm.201607291

**Published:** 2017-03-09

**Authors:** Annelien L Bredenoord, Insoo Hyun

**Affiliations:** ^1^ Department of Medical HumanitiesJulius CenterUniversity Medical Center UtrechtUtrechtThe Netherlands; ^2^ Department of BioethicsCase Western Reserve University School of MedicineClevelandOHUSA

**Keywords:** Genetics, Gene Therapy & Genetic Disease, Stem Cells, Urogenital System

## Abstract

A timely and thoughtful discussion of the ethical challenges and societal impacts presented by the recent reports demonstrating the successful *in vitro* generation of functional gametes from somatic and pluripotent stem cells.

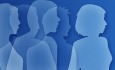

The year 2016 gave a glimpse of a future where functional gametes can be made in a dish. Pursuit of the creation of oocytes and sperm from stem cells broadly serves two ends: (i) scientific research, for example, understanding infertility or gametogenesis and (ii) new assisted reproductive technology (ART) development. Creating sperm and oocytes *in vitro* may well provide revolutionizing options for both research and reproduction, but is replete with ethical and societal challenges. Here, we discuss some of the key ethical challenges and promises.

## Introduction: why create gametes in a dish?

Last year, several research groups made important advances toward the *in vitro* creation of gametes after preliminary research had indicated that gametes could be derived from somatic and pluripotent stem cells (Hayashi *et al*, 2012). In 2016, Japanese researchers generated the first functional male and female mouse germ cells. They reprogrammed mouse skin cells into induced pluripotent stem cells and then into oocytes. By way of *in vitro* fertilization (IVF), 26 new mice were born from these oocytes, some of which gave birth to mouse pups themselves (Cyranoski, 2016; Hikabe *et al*, 2016). Another group created spermatid‐like cells from embryonic stem cells and primordial germ cells, which resulted in viable and fertile offspring (Zhou *et al*, 2016). Creating oocytes and sperm in the laboratory broadly serves two ends: scientific research, for example, to understand infertility or gametogenesis and the development of new assisted reproductive technologies (ART).

Fortunately, most couples can have children “the usual way,” but some couples, which suffer from subfertility, require ART to have children. These prospective parents can use IVF or intracytoplasmic sperm injection (ICSI), both of which depend on the premise that both partners produce viable gametes (Ishii & Pera, 2016). There are other people, however, who need a gamete donor to have children (Hendriks *et al*, 2015): heterosexual couples of reproductive age if one of the partners is infertile due to chemotherapy, genetic causes, injuries or infections; menopausal woman; same‐sex couples (both male and female); and singles. If sufficiently safe and effective, stem cell‐derived gametes might in the future provide an option for these people to have genetically related children. It might also enable other types of family constructions.

For now, the technique to generate gametes *in vitro* is far away from being sufficiently safe and effective and clinical applications remain remote, which gives us time to understand and evaluate the ethical and societal implications of this new technology. Indeed, the creation of stem cell‐derived gametes for both scientific research and human reproduction raises novel ethical questions, and revives ethical challenges from earlier discussions on ART.

## Ethical aspects in basic research

First, basic and preclinical research to generate gametes *in vitro* will involve the creation, use, and destruction of oocytes and embryos. For example, stem cell‐derived oocytes need to undergo functional tests using natural sperm, which creates embryos. Similar to research that enabled mitochondrial replacement techniques, it will create new life and potentially germline modifications. Although diverse views exist on whether it is morally justified to create and use human embryos for research—both intentionally and as a side effect—many countries have adopted a gradualist position that grants the early embryo some moral standing which increases throughout development and pregnancy. This position has resulted in the 14‐day rule that permits research on human embryos to the point where the primitive streak appears, the point in development after which embryos cannot split into twins or fuse together anymore (Hyun *et al*, 2016). It also represents the earliest development of the nervous system. Nevertheless, many of these countries do not allow the deliberate creation of human embryos for research while some countries, such as the USA, do not oversee such activities at all (Hyun, 2014).

We consider a ban on creating embryos for research to be morally problematic, since widely accepted IVF procedures for reproduction also involve the creation and discarding of surplus or unsuitable human embryos for a medical treatment, namely to treat infertility. This sends a message that the creation and destruction of embryos for infertility is more acceptable than the creation of embryos for research to develop new therapies for infertility and other common and often lethal medical conditions (Bredenoord *et al*, 2017).

Second, any initial clinical use of stem cell‐derived gametes will be replete with risks and uncertainties. Therefore, thorough and extensive preclinical research on human embryos is required to improve safety and efficacy. It would, for instance, be necessary to examine in preclinical assays whether research embryos develop normal body plans and germ layer formation compared to control embryos. This might eventually require culturing research embryos *in vitro* up to and beyond 14 days. The 14‐day rule is a practical and legal line in the sand and has served well as a policy tool to balance research ends with the moral standing of embryos. It will be valuable and potentially necessary—also because of other scientific developments such as organoid research—to revisit the ethical arguments behind the 14‐day rule and relevant regulations (Hyun *et al*, 2016; Bredenoord *et al*, 2017).

Third, international stem cell research ethics dictates that human biological material must be obtained in an appropriate manner (Daley *et al*, 2016). A key principle is that human biomaterials used to derive new stem cell lines must be obtained with explicit and voluntary informed consent by the donor, and that consent is consistent with the proposed research use of the material. Contrary to this high standard, it is possible to obtain human biological material for research without such specific consent in some countries. For example, US federal research regulations permit research involving “pathological specimens, or diagnostic specimens, if these sources are publicly available or if the information is recorded by the investigator in such a manner that subjects cannot be identified, directly or through identifiers linked to the subjects” (US Government 2009, *Code of Federal Regulations* Title 45, Part 46.101.b.2. http://www.hhs.gov/ohrp/sites/default/files/ohrp/policy/ohrpregulations.pdf). This means that, without local policies that would impose more stringent requirements, any tissues discarded during clinical practice can be used for this type of research without explicit consent of the patient, as long as the tissues are anonymized and the patient's admission or consent form for diagnostic or surgical procedures states that biomaterials collected during treatment may be used for “education and research”. This highlights the importance of and challenge for scientists to ensure the ethical provenance of human biomaterials used for the derivation of induced pluripotent stem cell lines that might be used to create gametes. Patients who are the genetic sources of these gametes may have no awareness of—let alone the opportunity to consider—whether they would explicitly want their clinically discarded tissues to be used for such research.

Fourth, creating stem cell‐derived gametes raises the exciting possibility of making oocytes for research. Oocyte donation for research purposes is an accepted practice in many countries, and the International Society for Stem Cell Research (ISSCR) has formulated conditions for donation (Daley *et al*, 2016). However, the procurement of oocytes involves risks, burdens, and inconveniences for the women, and there has been a long‐standing shortage of research oocytes. Stem cell‐derived gametes may therefore be a long‐awaited alternative. However, an easy supply of oocytes may result in the creation of a large number of research embryos on a scale currently unthinkable owing to the scarcity of oocytes for research, a situation that is sometimes pejoratively referred to as “embryo farming” (Cohen *et al*, 2017). This minimally requires specific oversight mechanisms and regulations for the creation and use of stem cell‐derived oocytes.

Fifth, stem cell‐derived gametes can be genetically modified using genome editing tools, such as CRISPR/Cas9, both for research and for preventing inheritable diseases, but also for enhancement. Although the ethics of germline genome editing is beyond the scope of this commentary, it is immediately clear that this application should be discussed along with the broader debate on the ethics of germline gene editing.

## Ethical aspects in human reproduction

First, stem cell‐derived gametes may offer reproductive options specifically for couples who were hitherto dependent on a donor to have children. This technology may, for example, provide an option for cancer survivors who lost fertility after chemotherapy, patients with genetic disorders, ovulation disorders, and many other causes of infertility in both men and women. Stem cell‐derived gametes may therefore help infertile couples to fulfill their desire to have children. In addition, it may have an emancipating and liberating effect for other people who are unable to have biological children altogether, such as same‐sex couples. In this sense, it may “democratize reproduction” (Testa & Harris, 2005) and could result in “fertility for everyone” (Smajdor and Cutas, 2015 Artificial gametes. Background paper for the Nuffield Council of Bioethics http://nuffieldbioethics.org/wp-content/uploads/Background-paper-2016-Artificial-gametes.pdf).

The most paradigm‐shifting use of stem cell‐derived gametes could be applications to enable more than two genetic parents to have a child together, also described as “multiplex parenting”. A single child might be brought into existence through a rapid succession of genetic generations. First, two *in vitro* embryos would be derived from two different couples either by IVF using their own gametes or by creating stem cell‐derived gametes. Subsequently, embryonic stem cell lines would be derived from each of these embryos and differentiated into gametes out of which a single new embryo would be created and gestated. In this—still hypothetical—case, four people would together create a child of which they are the genetic grandparents, thus perhaps embodying their wanted sense of genetic kinship with the offspring (Palacios‐Gonzales *et al*, 2014).

This and other applications will inevitably trigger “this is unnatural” type of objections, or appeals on the “yuck factor,” which have been proven flawed and morally prejudiced in earlier discussions (Nussbaum, 2006). For this reason, it would be prudent to avoid terms such as “artificial” or “synthetic” gametes, as these labels may give the pejorative impression that stem cell‐derived gametes are ethically inferior to other types of ART. There is a need, however, for ethical and legal research to evaluate these new constructions of modern families—in many countries, the nuclear family is still entrenched in the law (Smajdor & Cutas, 2014).

Moreover, this emerging technology requires sociological research to evaluate the long‐term welfare of children born through these techniques. It is widely acknowledged that the welfare of the child is an important moral consideration, specifically in ART. Earlier we, and others, defended the reasonable welfare standard: that to justify medically assisted reproduction, the child‐to‐be must have a reasonable chance of an acceptable quality of life (Bredenoord *et al*, 2008). This, of course, needs further, extensive elaboration for the specific circumstances of the new technique, but may serve as a rule of thumb for assessing the welfare of future children conceived this way.

Second, the creation of stem cell‐derived gametes may exert social pressure on infertile patients to use this technology to have their “own children” and may aggravate a one‐sided focus on having genetically related children (Illioi & Golombok, 2015). But, if we take this argument seriously, we should also apply it to IVF practices. Apparently, people generally prefer having biological children of their own and consider other options only when this becomes difficult or impossible. Moreover, the decision whether and how to have children could be considered to a certain limit as part of a couple's reproductive autonomy. Nonetheless, we should continuously monitor the social value of infertility research, for instance whether it is proportionate to invest limited financial and scientific resources in increasingly refined fertility research (Cutas *et al*, 2014; Hendriks *et al*, 2015).

Third, if gametes can be made out of skin cells, this could not only result in unwitting research donors as discussed above, but also in hypothetical unwitting parents when “donors” do not know at all that gametes have been made out of their tissues (Smajdor & Cutas, 2014). Although problematic, this concern might be mitigated by legal and regulatory measures for making and using stem cell‐derived gametes. It would place significant responsibilities on professionals and parents using ART with stem cell‐derived gametes.

## Conclusion

The year 2016 saw the first tentative steps toward making functional gametes *in vitro*. Creating sperm and oocytes out of stem cells may provide revolutionizing options for both research and reproduction, but it is replete with ethical and societal challenges. The generation of stem cell‐derived gametes is in the foreseeable future and will raise fierce ethical, societal, and political debates around the world. The history of reproductive technologies shows that innovative reproductive techniques are often introduced without a sound evaluation (Dondorp & De Wert, 2011). While preclinical and basic research progresses, we need to pay more attention to the ethics of research *per se* and the ethical, legal, and social implications of new family constructions. Specific legal and regulatory measures and conditions will be needed for making and using stem cell‐derived gametes. Responsible innovation requires a commitment from all parties involved, to address in a timely fashion the scientific, ethical, and legal challenges of *in vitro*‐derived gametes in research and reproduction.
